# The Landscape of Clinical Trials in Never-Smoker Non-Small-Cell Lung Cancer: Registered Evidence and Persistent Gaps

**DOI:** 10.3390/cancers18040551

**Published:** 2026-02-08

**Authors:** Raquel Ramos, Carlos Sousa, Nuno Vale

**Affiliations:** 1 PerMed Research Group, RISE-Health, Faculty of Medicine, University of Porto, 4200-319 Porto, Portugal; raquelramos@med.up.pt (R.R.); carlos.sousa@synlab.pt (C.S.); 2 RISE-Health, Department of Community Medicine, Health Information and Decision (MEDCIDS), Faculty 12 of Medicine, University of Porto, Rua Doutor Plácido da Costa, 4200-450 Porto, Portugal; 3 Molecular Biology Department, SYNLAB Portugal, Rua Manuel Pinto de Azevedo, 401, 4100-321 Porto, Portugal; 4 Laboratory of Personalized Medicine, Department of Community Medicine, Health Information and Decision (MEDCIDS), Faculty of Medicine, University of Porto, 4200-450 Porto, Portugal

**Keywords:** lung cancer, never-smokers, non-small cell lung cancer, clinical trials, ClinicalTrials.gov, molecular profiling, biomarker-driven therapy, targeted therapy, precision oncology

## Abstract

Lung cancer in never-smokers is increasingly recognized as a distinct clinical and biological entity, often enriched for actionable oncogenic alterations and characterized by molecular profiles that differ from tobacco-associated disease. However, therapeutic evidence for this growing patient population has frequently been extrapolated from trials in which smokers predominate, potentially limiting treatment optimization and biomarker-driven decision-making. In this review, we map and critically appraise clinical trials registered in ClinicalTrials.gov that explicitly target never-smokers with lung cancer, focusing on therapeutic strategies, molecular stratification approaches, trial design features, and temporal trends. We discuss how eligibility definitions, histological and genomic enrichment, and endpoints have been handled across studies, and we highlight persistent gaps in dedicated trial activity, particularly in prospective biomarker-guided designs and contemporary platform strategies. Finally, we propose priorities for future trials to better reflect never-smoker lung cancer biology, improve external validity, and accelerate evidence generation for personalized therapeutic approaches in this population.

## Simple Summary

An increasing number of people are developing lung cancer despite never having smoked. These tumours often behave differently and may carry specific molecular changes that can potentially guide treatment. Yet, much treatment evidence has historically been derived from trials that mainly include smokers, which may not fully represent never-smokers. In this review, we examine registered clinical trials that were specifically designed for never-smokers with lung cancer. We summarize what types of treatments were tested, how patients were selected, whether molecular testing was used to guide enrolment, and how trial activity has evolved over time. We also identify key gaps and propose priorities for future studies so that never-smokers with lung cancer can benefit from evidence generated in trials tailored to their condition. Addressing these gaps is essential to ensure that never-smoker lung cancer patients benefit from evidence truly reflective of their disease biology.

## 1. Lung Cancer in Never-Smokers: An Emerging Entity

Lung cancer (LC) is the most prevalent cancer worldwide, with 2022 estimates reporting approximately 2.5 million new cases and 1.8 million deaths [1]. It remains one of the most complex cancers, being characterized by a late diagnosis and poor outcomes due to its non-specific symptoms—cough, dyspnea, fatigue, chest pain, and weight loss [2]. As is already known, tobacco smoking is the strongest risk factor for LC development, accounting for nearly 80% of cases. Nonetheless, there are other causes for LC development not related to tobacco [3].

Histologically, LC is a heterogeneous disease that can be divided into two major groups: non-small-cell lung cancer (NSCLC) and small-cell lung cancer (SCLC) [4]. Within these groups, SCLC is strongly associated with tobacco smoking, comprising 10–15% of all lung cancers. Otherwise, NSCLC is the most common lung cancer type, representing about 85% of cases. Among its subtypes, adenocarcinoma is the most common, occurring frequently among non-smoking females [5,6].

These histological and molecular differences have major therapeutic implications, supporting the shift toward biomarker-driven decision-making and, consequently, distinct patient outcomes and therapeutic approaches. In particular, once NSCLC is histologically classified, it should be further evaluated through multiple molecular methods, such as fluorescence in situ hybridization (FISH), immunohistochemistry (IHC), next-generation sequencing (NGS), and real-time polymerase chain reaction (RT-PCR) to detect genetic alterations with known targeted therapies [7,8]. Over the past decade, traditional chemotherapy and radiotherapy have been increasingly complemented and, in many cases, replaced by immunotherapy and targeted therapies directed at specific molecular alterations. These agents target identifiable oncogenic drivers or biomarkers, enabling more precise and effective treatment strategies while reducing the toxicity associated with conventional approaches [9]. Moreover, these genetic alterations are different between smokers and never-smokers, which suggests different mechanisms of tumour development in these patients (Table 1) [10,11]. Specifically, tumours arising in never-smokers commonly harbour *EGFR* mutations, mainly L858R in the exon 21 mutation. Also, gene fusions involving *ALK*, *ROS*, or *PIK3CA* were identified as being more common in never-smokers. Conversely, *KRAS* mutations occur less frequently in this population compared with tumours from smokers [11,12,13,14].

Moreover, recent research indicates that lung adenocarcinoma (LUAD) arising in never-smokers is characterized by a higher frequency of clinically actionable driver mutations than LUAD associated with smoking (78–92% versus 49.5%). Furthermore, RNA-sequencing analyses have identified distinct immune-related transcriptional subtypes in never-smoker LUADs, which differ in both the expression of therapeutically relevant immune checkpoint molecules and the composition of infiltrating immune cell populations [15]. At the same time, epigenetic mechanisms are increasingly recognized as key factors in differentiating NSCLC in smokers and never-smokers. A recent investigation provided an in-depth comparison of the epigenomic landscapes of these two patient groups, focusing on the role of DNA methylation alterations in shaping tumour phenotypes. The study identified recurrent promoter methylation changes associated with smoking status. Notably, tumours from never-smokers exhibited a higher prevalence of hypomethylated differentially methylated regions (hypoDMRs) and a greater number of consistently hypomethylated gene promoters, including ASPSCR1, TOP2A, DPP9, and USP39, previously implicated in cancer development [16].

As stated before, tobacco smoking is not the single determinant of lung cancer development, as multiple environmental and genetic factors also contribute. In reality, other risk factors are associated with this disease, namely environmental and occupational exposure, family history of lung cancer (genetic susceptibility), hormonal factors, and previous lung diseases [17,18,19]. In fact, the incidence of LC in never-smokers is around 20% and is increasing. Recent data indicate that the proportion of cases occurring in never-smokers has increased to approximately 17% in men and 24% in women [11,13].

Therefore, considering this increase in LC cases in never-smokers and the biological differences between smokers and never-smokers, it is obvious that the traditional focus on smoking is no longer enough. Accordingly, this article focuses on LC in people who have never smoked, investigating its unique characteristics, available therapies, and the respective current status of clinical trials in order to identify gaps and future directions for these patients.

## 2. Evolution of Therapeutic Clinical Trials in Never-Smokers

As with other diseases, several clinical trials have been conducted in LC to evaluate novel therapeutic strategies and improve patient outcomes. Over the past years, more attention has been given to the distinct clinical and biological characteristics of LC arising in never-smokers. Consequently, various clinical trials have been specifically designed to investigate therapeutic options in the never-smoker population, recognizing this group as a distinct clinical entity. Table 2 summarizes all the therapeutic clinical trials conducted in the past years that exclusively enrolled never-smokers with LC. The search was conducted in ClinicalTrials.gov without date restrictions. Only interventional studies were included in the analysis to examine the evolution of therapeutic strategies in this patient population, while observational and epidemiological studies were excluded. The search strategy used a combination of terms including “lung cancer”, “non-smoker”, and “never-smoker”.

All the studies presented before evaluated a range of therapeutic agents and molecular targets for a specific population of LC patients, carefully selected according to rigorous eligibility criteria. According to ClinicalTrials.gov, those criteria are similar among the studies and mainly include a confirmed diagnosis of NSCLC (locally advanced or metastatic disease), 18 years and older, and never-smokers (≤100 cigarettes in lifetime) or former light smokers (who smoked between >100 cigarettes and ≤10 pack-years and quit ≥1 year ago). Nonetheless, each trial presents some specific criteria, especially related to genetic characteristics and drug sensitivity. Specifically, some trials testing tyrosine kinase inhibitors (TKIs), namely erlotinib and gefitinib, in combination with other drugs (such as pemetrexed and bevacizumab) took into account *EGFR* mutations and/or tumour sensitivity to TKIs [20,25]. Another clinical trial (NCT01829217) selectively enrolled patients whose tumours were wild-type for *EGFR*, *KRAS*, and *ALK* or harboured a *RET* rearrangement, given sunitinib’s inhibitory activity against *RET* [24].

According to Table 2, more than half of the trials test an EGFR inhibitor—gefitinib or erlotinib—in monotherapy or in combination with an antiangiogenic drug (bevacizumab). In fact, most of them test EGFR inhibitors alone, which may reinforce the importance of TKIs in the treatment of LC. On the other hand, targeting angiogenesis also seems to be a promising therapeutic strategy, as approximately half of the studies evaluate antiangiogenic drugs. Among these, four studies investigate sorafenib or sunitinib in monotherapy. Sunitinib is currently approved for renal cell carcinoma (RCC), gastrointestinal stromal tumours (GISTs), and pancreatic neuroendocrine tumours, targeting vascular endothelial growth factor receptor 1 (VEGFR1) 1, VEGFR2, VEGFR3, PDGFR-α, PDGFR-β, fibroblast growth factor receptor 1 (FGFR1), fms-related tyrosine kinase 3 (FLT3), stem cell factor receptor (c-Kit), RET, MET proto-oncogene, and colony-stimulating factor 1 receptor (CSF1R) [32]. Similarly, sorafenib is primarily used to treat renal and liver cancer, and its mechanism of action involves inhibiting tumour angiogenesis and cell proliferation by targeting VEGFR and Raf kinase [33]. Although neither sunitinib nor sorafenib is approved for the treatment of LC, several of the molecular pathways targeted by these agents are known to be active in this cancer, particularly in NSCLC, such as VEGFR and the aberrant activation of RAF/MEK/ERK [34,35]. Therefore, the evaluation of sorafenib and sunitinib in lung cancer can reasonably be considered an example of drug repurposing, as these agents are being investigated outside their original approved indications based on shared oncogenic and angiogenic mechanisms.

**Figure 1:**
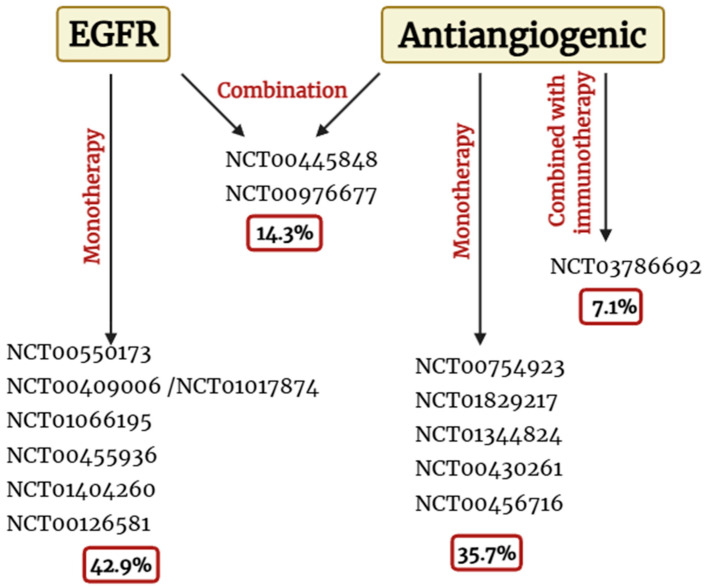
Distribution of clinical trials according to therapeutic target and treatment strategy in lung cancer. The figure summarizes the clinical trials previously identified, classified according to the main therapeutic target (EGFR or angiogenesis) and the treatment strategy employed (monotherapy or combination with other agents, including immunotherapy). The percentages indicate the proportion of clinical trials targeting a specific pathway relative to the total number of clinical trials analyzed, as well as their distribution across the different therapeutic approaches. Trials in which the combination involved only conventional chemotherapeutic agents and no other targeted therapies were included in the monotherapy group. Created in https://BioRender.com (27 January 2026).

Thus, considering the previous analysis, it is understandable that EGFR-targeted and antiangiogenic drugs were the main therapeutic focuses of these clinical trials. This fact may be justified by EGFR’s biological relevance in LC and due to the overall dependence of the tumour on angiogenesis in all molecular subtypes. Consequently, the clinical trials can be divided into the subgroups shown in Figure 1.

Nonetheless, despite the promising research conducted in this area and the generally encouraging results obtained, most of the clinical trials are relatively old, having been conducted between 2010 and 2015. This time window coincides with the early adoption of EGFR testing and the first wave of targeted therapy approvals in NSCLC, which largely shaped research priorities during that decade. A smaller number of studies were carried out between 2017 and 2019. However, the most recent trials are NCT00445848 and NCT03786692, conducted in 2020 and 2025, respectively. This fact, combined with the small number of clinical trials performed in this specific group of patients (only 14), highlights the limited research in this area. The concentration of studies conducted between 2010 and 2015 may be related to the period during which an increasing incidence of LC among non-smokers was first recognized, leading to the understanding that this population represents a distinct clinical and molecular subgroup. Conversely, the low number of trials in other years may be due to the historically small size of this patient group, which makes research more challenging and data more limited. Also, the historical focus of lung cancer research on smoking-associated disease may have reduced prioritization and funding for trials targeting never-smokers. However, in recent years, the incidence of lung cancer among never-smokers has been rising, emphasizing the growing need for targeted research in this population. Beyond smoking status, the integration of biomarker-driven enrollment has become crucial for including never-smokers, who are often enriched for actionable molecular alterations such as *EGFR* or *ALK*, allowing for their participation in clinical trials.

## 3. Perspectives and Future Directions—What Comes Next?

Despite some important steps forward, existing trials in never-smoker lung cancer remain fragmented and outdated, lacking the coherence and innovation seen in broader NSCLC research. The evidence presented in Section 2 showed us that despite some efforts in improving the therapeutic options for never-smoker LC patients, these attempts are older, disorganized, and lacking in a coherent strategy. Even today, most clinical trials in LC are conducted either in smoker populations or without accounting for smoking history, treating all lung cancer patients as a homogeneous group. However, it is well established that LC is a heterogeneous disease, and smoker and never-smoker patients must be treated as different entities. Accordingly, clinical trials should be designed to reflect this distinction.

As mentioned, the number of never-smoker LC patients has increased over the years, as well as the number of deaths, with this disease being the current fifth most common cause of cancer-related deaths worldwide [18]. Consequently, it is urgent to design and conduct new clinical trials specifically targeting never-smokers to better understand their tumour behaviour and discover new possible targeted therapies to improve their outcomes. On the other hand, despite this need, there remains a critical gap in the understanding of tumour genetics and molecular features in this specific population. As stated in Section 1, some genetic alterations are known to be more present in never-smokers than in smokers. However, to fully elucidate these differences and the molecular mechanisms, more translational research is needed, including comprehensive molecular profiling and multi-omics analyses—including genomics, transcriptomics, proteomics, metabolomics, and epigenomics. Such efforts may lead to the identification of novel actionable targets, the validation of predictive biomarkers, and guide biomarker-based clinical trial design. Collaboration between institutions is also crucial for faster results. Ultimately, integrating these insights into clinical trial design could optimize patient selection, improve therapeutic efficacy, evaluate new or repurposed drugs beyond the conventional EGFR and antiangiogenic therapies explored in earlier trials, and accelerate the development of personalized treatment strategies for never-smoker LC patients.

On the other hand, a study performed in a large NSCLC cohort showed that never-smokers were more likely to be diagnosed at an earlier stage (stage I–II) compared with smokers, suggesting differences in clinical presentation between these groups. In this scenario, surgical resection remains the mainstay of curative treatment [7,36]. The different molecular profiles and survival statuses between smokers and never-smokers may influence postoperative risk stratification and adjuvant treatment decisions. Therefore, future perioperative and adjuvant clinical trials specifically designed for never-smokers may help refine recurrence risk assessment and guide personalized adjuvant or neoadjuvant therapeutic strategies in early-stage disease.

Complementarily, since NSCLC is the most common lung cancer subtype in never-smokers, it is understandable that clinical trials conducted to date have primarily focused on this histology. However, building on the improved molecular and genetic characterization efforts discussed above, it would be of interest to investigate the occurrence of SCLC in never-smokers and to explore therapeutic strategies specifically directed at this subtype.

Furthermore, in silico and computational approaches are excellent tools to improve our understanding of LC in never-smokers and to predict therapy responses. Some studies have already developed risk prediction models specifically for this population. Those are crucial to access personalized risk assessment for lung cancer, helping identify high-risk never-smoking patients and helping in the formulation of future LC screening strategies, where the inclusion of this population is vital [37]. Nevertheless, it remains important to continue research in this area to refine predictions and integrate them with molecular and therapeutic data. Moreover, in silico research focused on never-smoker LC patients remains more centred on these computational risk prediction models. Consequently, other advanced studies such as therapy response modelling, virtual trials, or even digital twin approaches are still largely unexplored in this population.

Considering the heterogeneity of lung cancer and the distinct molecular signatures in never-smokers, digital twin technology may serve as a dynamic integrative platform to simulate tumour evolution, optimize therapy, and advance precision oncology. Digital twin technology is an emerging computational approach that enables the simulation of disease progression and treatment responses through the integration of multi-level patient data. In oncology, this field is still in early development but shows potential for supporting clinical decision-making, optimizing therapeutic strategies, and guiding the design of future clinical trials. However, several challenges remain, including data heterogeneity, technical complexity, computational requirements, and ethical considerations [38,39]. Nevertheless, as the molecular and clinical characterization of lung cancer in never-smokers continues to improve, digital twin-based approaches may in the future offer a complementary framework to explore disease behaviour and personalized therapeutic strategies in this patient population.

## 4. Conclusions

Lung cancer remains the most prevalent and deadliest cancer worldwide. Over recent years, the epidemiological profile of this disease has been changing, with a growing proportion of cases occurring in never-smokers. This trend partly reflects the global decline in tobacco consumption but also indicates the increasing contribution of other risk factors such as air pollution (e.g., long-term exposure to PM_2.5_), occupational exposures, and genetic susceptibility. Non-small-cell lung cancer (NSCLC) is the predominant type among never-smokers, whose tumours often display distinctive genetic and molecular features. Consequently, the design of therapeutic regimens tailored to these molecular characteristics is of paramount importance. Never-smoker lung cancer frequently harbours oncogenic drivers (e.g., EGFR, ALK, ROS1, RET, MET). It exhibits a distinct immune–genomic milieu compared with tobacco-associated disease, with implications for both targeted therapies and immunotherapy. These features underscore the need for comprehensive molecular profiling at diagnosis, including broad next-generation sequencing panels, optimizing therapeutic matching, and avoiding under-treatment. In parallel, improved phenotyping of environmental exposures, particularly fine particulate matter and indoor/occupational toxicants, should be integrated into clinical datasets to clarify exposure–genotype–phenotype relationships and refine risk stratification [40,41,42].

Although some clinical trials have specifically addressed never-smoker patients, most remain limited in scope and focus mainly on classical targets such as EGFR and angiogenesis. Future studies should adopt biomarker-enriched umbrella/basket designs, adaptive randomization, and pragmatic endpoints that reflect real-world effectiveness. Harmonized definitions of “never-smoker”, standardized exposure assessment, and better representation of women and diverse geographic regions will also be critical to improve external validity and equity of evidence. Given the rising incidence and mortality within this group, there is an urgent need to reinvigorate research efforts through the integration of multi-omics approaches (genomics, transcriptomics, proteomics, and metabolomics), interinstitutional collaboration, and in silico methods. Mechanistic and data-driven models can accelerate target discovery, drug repurposing, and dose optimization, while federated analytics may enable privacy-preserving learning across institutions. In parallel, investment in digital twin technologies, namely virtual, continuously updated patient models that couple tumour biology, pharmacology, and clinical trajectories, represents a promising and underexplored strategy to forecast response, personalize sequencing of therapies, and improve outcomes for never-smoker lung cancer patients.

Finally, a comprehensive agenda should align prevention and therapy, including strengthening air quality regulations and workplace safety, deploying early-detection strategies tailored to never-smokers with high non-tobacco exposure burden, and embedding longitudinal exposure metrics into clinical care. Such an integrated framework, linking exposure mitigation, precision diagnostics, and adaptive therapeutics, will be essential to bend the mortality curve for never-smoker lung cancer.

## Figures and Tables

**Table 1:** Prevalence of major genetic alterations in lung cancer according to smoking status [13].

Mutation/Alteration	Smokers (%)	Never-Smokers (%)
EGFR	15.6%	21.5%
KRAS	16.3%	11.5%
PIK3CA	6.8%	9.5%
ALK	3.2%	7.5%
MAP2K1	6.6%	3.5%
MET	3.4%	4.5%
ROS1	0.6%	2.5%

**Table 2:** Clinical trials in never-smoking lung cancer patients, showing year of last update, study phase, recruitment status, tested drugs, their targets, and the respective results.

Clinicaltrials.gov Identifier	Year of Last Update	Phase	Recruitment Status	Tested Drug(s)	Targets	Results	Ref
NCT00550173	2013	II	Completed	Pemetrexed + Erlotinib	EGFR	PFS: 7.4 months for Erlotinib + Pemetrexed, 3.8 months for Erlotinib, and 4.4 months for Pemetrexed; combination therapy significantly improved PFS compared to either drug alone	[20]
NCT00409006 */NCT01017874 *	2010/2015	II/III	Completed/Completed	Chemotherapy + Gefitinib vs. Gefitinib monotherapy	EGFR	PFS: 8.3 months for gefitinib + chemo versus 9.6 months for gefitinib alone; OS: 26.9 months for gefitinib + chemo versus 27.9 months for gefitinib alone (PFS and OS were not significantly different between treatment arms); gefitinib was not efficacious in patients with wild-type EGFR; identification of EGFR mutation status is essential for NSCLC therapy	[21,22]
NCT00754923	2019	II	Terminated	Sorafenib	Antiangiogenic	PFS in 6 months: 0% and OS: 8.8 months; did not demonstrate significant long-term control of the disease; OS was low, indicating limited effectiveness	[23]
NCT01829217	2018	II	Completed	Sunitinib	Antiangiogenic	ORR: 7.7–8% (only 1 of 13 evaluable patients experienced a partial response); generally limited antitumour activity	[24]
NCT00445848	2020	II	Completed	Erlotinib + Bevacizumab	EGFR and Antiangiogenic	ORR: 50%; PFS: 7.4 months; OS: 29.8 months; therapy combination showed a significant efficacy in never-smoker advanced NSCLC	[25]
NCT01344824	2017	II	Completed	Carboplatin + Pemetrexed + Bevacizumab	Antiangiogenic	PFS: 12.6 months; ORR: 47%; OS: 20.3 months; combination showed activity with acceptable toxicity in these patients	[26]
NCT03786692	2025	II	Recruiting	Carboplatin + Pemetrexed + Bevacizumab + Atezolizumab	Antiangiogenic and PD-1/PD-L1 inhibition	No results posted	[27]
NCT01066195	2010	III	Unknown status	Gefitinib (experimental) vs. Pemetrexed (comparator)	EGFR	PFS: 9.0 vs. 3.0 months; ORR: 58.8% vs. 22.4%; No statistically significant difference in OS; gefitinib showed superior efficacy to pemetrexed as second-line therapy	[28]
NCT00430261	2010	II	Completed	Sunitinib	Antiangiogenic	No results posted	[23]
NCT00455936	2010	III	Completed	Gefitinib vs. Standard Chemotherapy (Gemcitabine Plus Cisplatin)	EGFR	OS: 22.3 vs. 22.9 (chemotherapy) months; 1-year PFS rate: 16.7% vs. 2.8% (chemotherapy); response rate: 55% vs. 46% (chemotherapy); gefitinib did not demonstrate superiority in terms of OS compared to chemotherapy	[29]
NCT00456716	2010	II	Completed	Sorafenib	Antiangiogenic	No results posted	[23]
NCT00976677	2014	II	Terminated	Carboplatin, Paclitaxel + Bevacizumab + Erlotinib Hydrochloride	Antiangiogenic and EGFR	PFS: 15.5 vs. 4.5 (control) months; more serious adverse events related to Erlotinib	[23]
NCT01404260	2017	III	Completed	Carboplatin + Gefitinib	EGFR	PFS: 9.7 vs. 4.2 (control) months; OS: 20.1 vs. 15.4 (control) months; adverse events were more common in the gefitinib arm	[30]
NCT00126581	2019	II	Completed	Erlotinib hydrochloride alone vs. Erlotinib Hydrochloride + Carboplatin + Paclitaxel	EGFR	PFS was similar (5.0 vs. 6.6 months); EGFR-mutant patients had a much better response	[31]

* Same clinical trial in different phases (II and III). OS—overall survival; PFS—progression-free survival; ORR—objective response rate.

## Data Availability

The original contributions presented in this study are included in the article material. Further inquiries can be directed to the corresponding author.

## Abbreviations

The following abbreviations are used in this manuscript:

c-KIT	Stem cell factor receptor
CSF1R	Colony-stimulating factor 1 receptor
FGFR1	Fibroblast growth factor receptor 1
FISH	Fluorescence in situ hybridization
FLT3	fms-related tyrosine kinase 3
GIST	Gastrointestinal stromal tumours
IHC	Immunohistochemistry
LC	Lung cancer
LUAD	Lung adenocarcinoma
NGS	Next-generation sequencing
NSCLC	Non-small cell lung cancer
ORR	Objective response rate
OS	Overall survival
PFS	Progression-free survival
RCC	Renal cell carcinoma
RT-PCR	Real-time polymerase chain reaction
SCLC	Small-cell lung cancer
TKIs	Tyrosine kinase inhibitors
VEGFR	Vascular endothelial growth factor receptor
